# Novel Cytochrome P450, *cyp6a17*, Is Required for Temperature Preference Behavior in *Drosophila*

**DOI:** 10.1371/journal.pone.0029800

**Published:** 2011-12-28

**Authors:** Jongkyun Kang, Jaeseob Kim, Kwang-Wook Choi

**Affiliations:** 1 Department of Biological Sciences, Graduate School of Nanoscience and Technology, Korea Advanced Institute of Science and Technology, Yuseong-gu, Daejeon, Korea; 2 Aprogen Inc., Yuseong-gu, Daejeon, Korea; VIB & Katholieke Universiteit Leuven, Belgium

## Abstract

Perception of temperature is an important brain function for organisms to survive. Evidence suggests that temperature preference behavior (TPB) in *Drosophila melanogaster*, one of poikilothermal animals, is regulated by cAMP-dependent protein kinase (PKA) signaling in mushroom bodies of the brain. However, downstream targets for the PKA signaling in this behavior have not been identified. From a genome-wide search for the genes regulated by PKA activity in the mushroom bodies, we identified the *cyp6a17* Cytochrome P450 gene as a new target for PKA. Our detailed analysis of mutants by genetic, molecular and behavioral assays shows that *cyp6a17* is essential for temperature preference behavior. *cyp6a17* expression is enriched in the mushroom bodies of the adult brain. Tissue-specific knockdown and rescue experiments demonstrate that *cyp6a17* is required in the mushroom bodies for normal temperature preference behavior. This is the first study, to our knowledge, to show PKA-dependent expression of a cytochrome P450 gene in the mushroom bodies and its role as a key factor for temperature preference behavior. Taken together, this study reveals a new PKA-Cytochrome P450 pathway that regulates the temperature preference behavior.

## Introduction

Behavioral responses to environmental stimuli, including light, humidity, and temperature, are important for survival of all living organisms. Especially for poikilothermal animals, extreme changes in ambient temperature or persistence for a long time at high or low temperature lead to death. These animals adapt to their body temperature changes by using molecular mechanisms to alter metabolism or by behavioral strategies to choose proper temperature conditions. *Drosophila* has been widely used as a genetic model for studying a variety of behaviors including learning. Recently, it has also been utilized to study the genetic basis of temperature sensation and temperature preference behavior.

In *Drosophila*, larvae and adult flies show strong temperature preference behavior [1]–[3]. A family of transient receptor potential (TRP) ion channels plays major roles in the sensation of temperature. For example, Painless, one of the TRP channel (TRPA1) superfamily, is required for sensing nociceptive stimuli over 38°C [4]. Another TRP channel, Pyrexia, is involved in protecting flies against noxious temperature over 40°C [5]. In contrast, *Drosophila* ANKTM1 TRP family channel participates in temperature selection by opening at warm temperature (24–29°C) [6], [7].

Despite extensive studies on the role of *Drosophila* TRP family channels in temperature sensing, it is not well understood how flies perform specific behavior to choose optimal temperature conditions. Interestingly, recent studies have shown that the mushroom bodies in the brain, which plays a critical role in learning and memory, is important for TPB [8]. Furthermore, cAMP-dependent PKA signaling in the mushroom bodies is not only essential for learning and memory but also for TPB. These studies have provided important clues to the mechanism underlying TPB, but the target genes for PKA signaling have been elusive. Hence, we carried out a genome-wide screen for the genes regulated by PKA to obtain insights into the molecular events underlying TPB. From this screen, we found *cyp6a17*, a cytochrome P450 superfamily gene, as a PKA downstream factor for TPB.

The cytochrome P450 (CYP) family is a diverse group of enzymes. Most CYP proteins are involved in the oxidation of a variety of organic substrates including natural products and detoxification of foreign compounds [9]–[14]. In *Drosophila*, there exist about 90 CYPs. Some of these CYP genes are involved in ecdysone hormones synthesis [15]–[20], male aggressive behavior [21], [22] and male mating [23]. However, no CYP genes have been implicated in specific brain functions like temperature sensing behavior.

Here, we show that *cyp6a17* is regulated by PKA and is required for temperature preference behavior. We demonstrate that *cyp6a17* expression in the mushroom bodies is necessary and sufficient for TPB. This study identifies *cyp6a17* as an important target of PKA signaling for mediating TPB in the mushroom bodies.

## Results

### Identification of new genes regulated by PKA in the mushroom bodies

**Figure 1:**
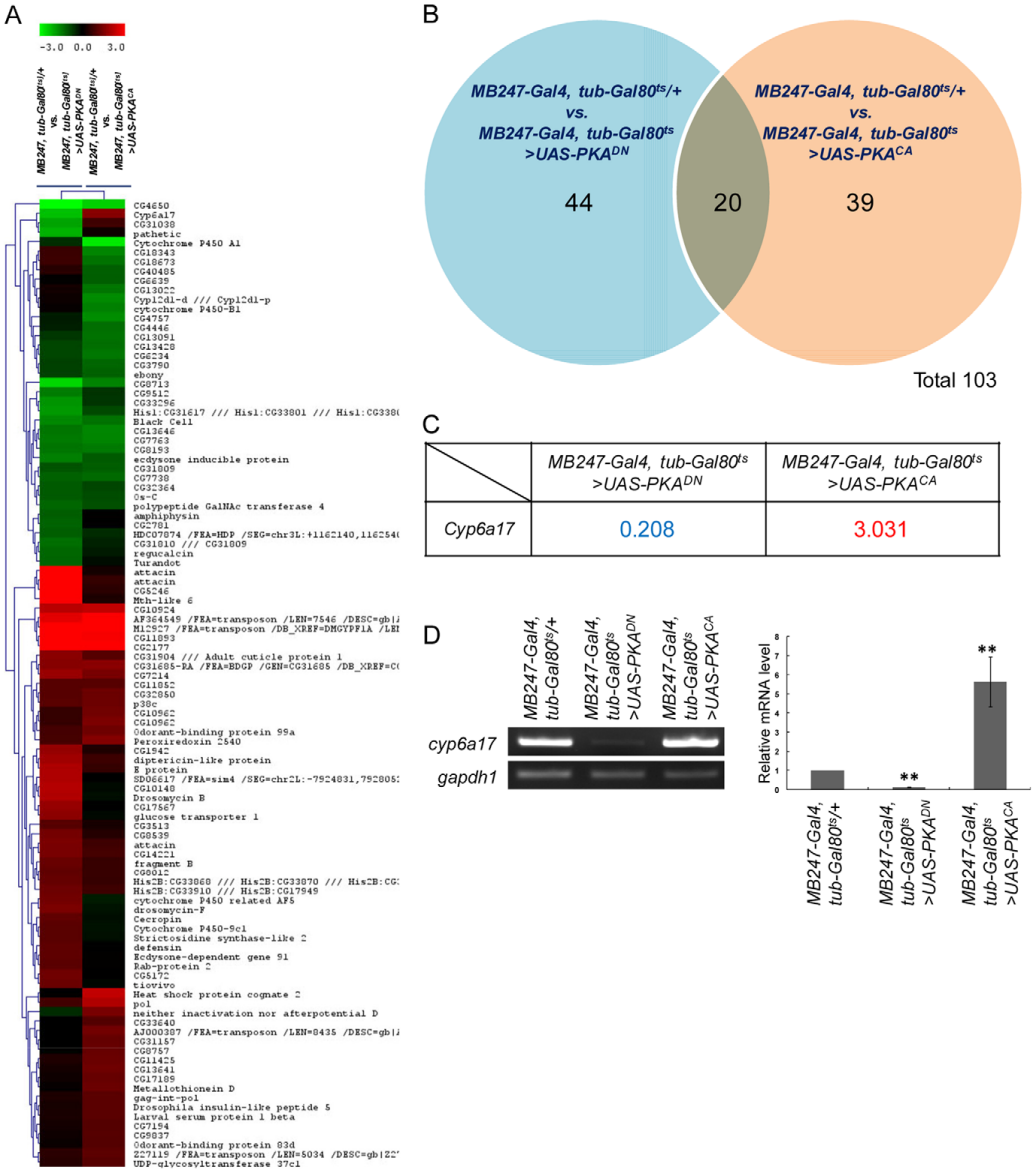
Genes differentially expressed by altered PKA activity in the mushroom bodies. (A) CLUSTER image of 130 PKA-regulated genes. Each column represents changes in the transcript level of candidate genes. Red indicates up-regulation by PKA^CA^, and green represents down-regulation by PKA^DN^. Identities of all PKA-regulated genes are provided on the right side of the CLUSTER image, placed in the same order as their relative position in the CLUSTER image. (B) Van diagram for 103 candidate genes. Blue area: 44 transcripts responded to PKA^DN^ but not PKA^CA^. Red area: 39 transcripts responded to PKA^CA^ but not PKA^DN^. Blue and red overlap area: 20 transcripts showed changes by both PKA^DN^ and PKA^CA^. (C) Transcript profiles of *cyp6a17* by PKA^DN^ or PKA^CA^. (D) RT-PCR confirmed the results of genome-scale microarray experiment. The relative mRNA transcript level was measured (histograms) by real-time qPCR analysis. *gapdh1* was used as internal control. The number of tests: N = 4. Two asterisks, *P*<0.001.

Temperature preference behavior in *Drosophila* depends on the level of PKA signaling in the mushroom bodies. To identify new components downstream to PKA, we carried out a genome-wide screen for genes regulated by PKA signaling in the mushroom bodies. Using the Gal4-UAS system, we increased or decreased PKA activity in the mushroom bodies by expressing dominant-negative (*UAS-PKA^DN^*) or constitutively active PKA (*UAS-PKA^CA^*), respectively. Expression of PKA transgenes was targeted to the mushroom bodies using the mushroom body-specific *MB247-Gal4* driver [8]. PKA expression was induced for 12–16 hours in three-day-old adults by inactivating the temperature-sensitive Gal80 [24] at the restrictive temperature. We then analyzed gene-expression profiles to identify the genes showing altered expression levels in response to the high or low PKA activity. The *Drosophila* GeneChip (DrosGenome 2.0) was used to obtain gene expression profiles from fly heads of three different groups: (i) the control group with no PKA transgene expression, (ii) the low PKA activity group and (iii) the high PKA activity group. Transcripts that showed more than 2-fold changes from the control expression level were considered for further analysis (Figure 1A & Table S1).

A total of 103 transcripts showing more than 2-fold changes were classified into three categories based on their responses to PKA activity: (i) 44 transcripts responded to PKA^DN^ but not by PKA^CA^. Among these, 14 showed downregulation while 30 resulted in upregulation. (ii) 39 transcripts showed altered expression by PKA^CA^ (23 increases and 16 decreases). (iii) 20 transcripts showed changes by both PKA^DN^ and PKA^CA^ conditions. Among them, 8 showed decreased and 11 showed increased transcript levels by both PKA^DN^ and PKA^CA^ conditions (Figure 1B).

Only one gene, *cyp6a17*, showed upregulation by PKA^CA^ and downregulation by PKA^DN^. We were most interested in this gene since its expression was correlated with both higher and lower PKA activities (Figure 1C). To confirm these microarray data, we examined the PKA-dependent expression of *cyp6a17* by RT-PCR and real-time-PCR. The expression level of *cyp6a17* mRNA in the head was decreased to 12% of the wild-type level by inhibiting PKA activity with mushroom body-specific expression of PKA^DN^. On the contrary, the transcript level was increased 5.63±1.32 times by PKA^CA^ overexpression (Figure 1D). Hence, *cyp6a17* expression in the head is regulated by PKA in an activity-dependent manner.

### *cyp6a17* mutations affect temperature preference behavior

**Figure 2:**
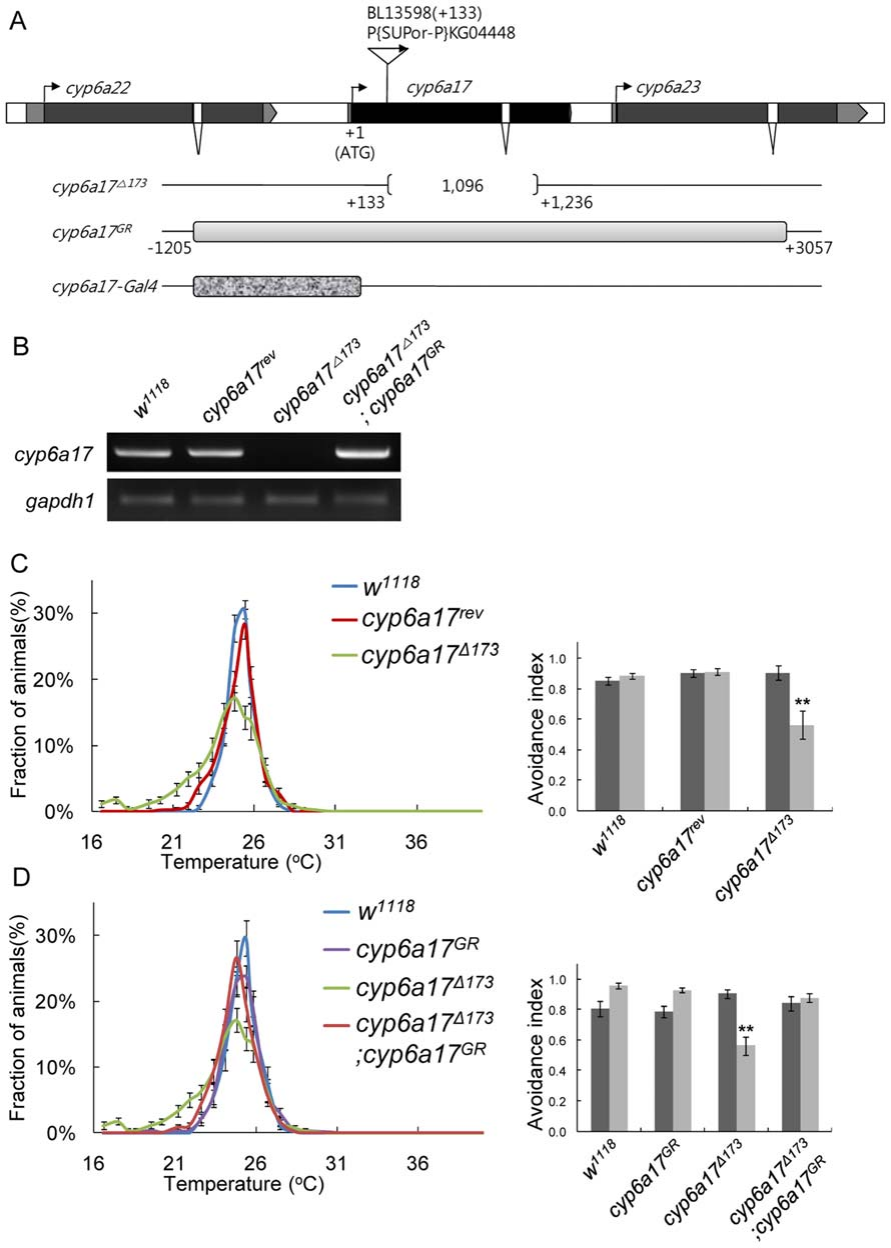
*cyp6a17* is required for normal temperature preference behavior. (A) Exons of *cyp6a17* are indicated by boxes, and coding regions are colored black. The P{SUpor-P} line has an insertion at 133 bp downstream from the translation start site. *cyp6a17^Δ173^* is a mutant with a deletion of 1.1 kb, as depicted by square brackets. A genomic DNA fragments used for rescue (*cyp6a17^GR^*) is indicated by gray box. The *cyp6a17-Gal4* construct contains a genomic fragment of 1.2 kb (short thick stippled box). (B) Determination of the expression level of *cyp6a17* by RT-PCR. *gapdh1* was used as internal control: N = 4. (C) *cyp6a17^Δ173^* showed reduced TPB. (D) The abnormal TPB of *cyp6a17^Δ173^* was restored by expression of the *cyp6a17* genomic transgene (*cyp6a17^GR^*). Black bars, AI_High_; gray bars, AI_Low_. One asterisk, *P*<0.005 and two asterisks, *P*<0.001.

To test whether *cyp6a17* is required for normal TPB, we analyzed the effects of *cyp6a17* mutations on TPB. We generated a deletion allele (*cyp6a17^Δ173^*) by inducing imprecise excision of the P-element *P{SUPor-P}KG04448* inserted at 133 bp downstream from the translation start site (Figure 2A). The *cyp6a17^Δ173^* mutation is a deletion of 1.1 kb sequence from the P-insertion site and affects only the *cyp6a17* gene (Figure 2A). Consistent with the deletion mapping, the transcript for *cyp6a17* was not detected in the *cyp6a17^Δ173^* mutant by RT-PCR analysis, suggesting that *cyp6a17^Δ173^* is a null mutation (Figure 2B). Homozygous *cyp6a17^Δ173^* mutant flies are viable and fertile with no visible morphological defects, suggesting that *cyp6a17* is not essential for normal development and fertility.

Next, we characterized the temperature preference in this deletion mutant. For TPB assays, all mutant and transgenic lines were outcrossed to the *w^1118^* line at least four times. *cyp6a17^Δ173^* mutant flies showed reduced AI (avoidance index) for low temperature (AI_Low_ = 0.56±0.06, *p*<0.001) (Figure 2C and Table S2). Immunocytochemcial analysis with anti-*FasII* antibody marker for the mushroom bodies did not reveal significant morphological defects in the brain of this mutant (Figure S1). Thus, the abnormal TPB shown in *cyp6a17* loss-of-function mutant is probably not caused by morphological defects in the mushroom bodies.

Specificity of the *cyp6a17* gene function in TPB was also confirmed by rescue of the *cyp16a17* mutant phenotypes. Abnormal TPB phenotypes of *cyp6a17* null mutant flies were rescued to the wild-type level by a 4.2-kb genomic DNA fragment (*cyp6a17^GR^* in Figure 2A) spanning the *cyp6a17* locus (AI_High_ = 0.84±0.04; AI_Low_ = 0.88±0.01) (Figure 2A, B, & D and Table S2). These data indicate that *cyp6a17* is required for normal TPB.

### Expression of *cyp6a17* is enriched in the mushroom bodies

To visualize the expression pattern of *cyp6a17*, we generated a *cyp6a17^MB^-Gal4* transgenic reporter strain in which Gal4 is induced by a *cyp6a17* genomic region containing the promoter. The translation start site for *cyp6a17* is only 612 bp apart from the adjacent upstream gene *cyp6a22*. *cyp6a17^MB^-Gal4* was created by cloning an 1,201 bp fragment upstream of the *cyp6a17* translation start site into pCaSpeR vector. This fragment contains the entire 5′ region of *cyp6a17* including a 3′ part of the adjacent *cyp6a22* gene. Therefore, the 5′ region of *cyp6a17* includes regulatory sequences necessary for the *cyp6a17* function in TPB (Figure 2A).

**Figure 3:**
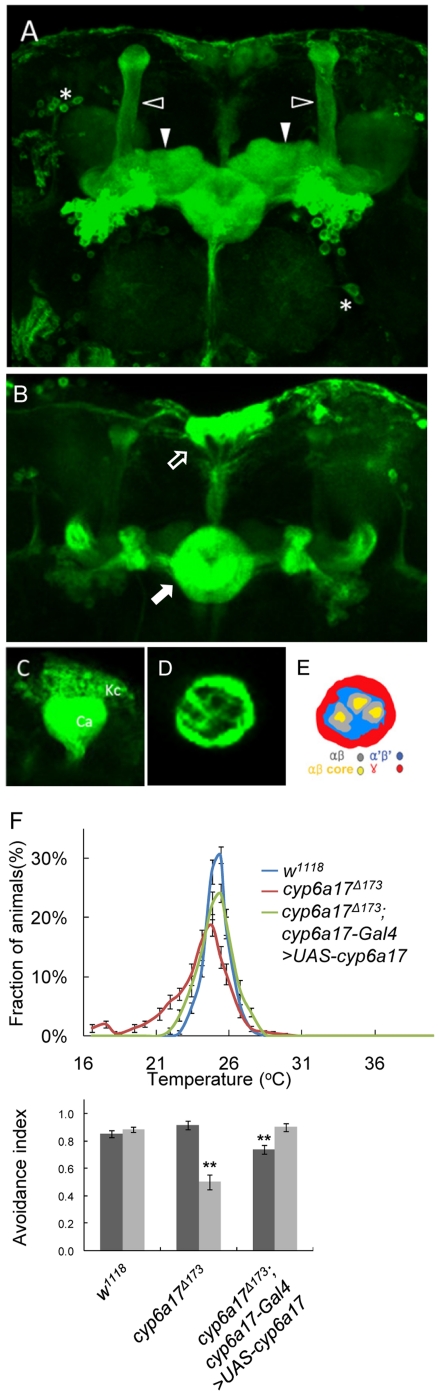
*cyp6a17* is expressed in the mushroom bodies. (A–E) *cyp6a17* reporter expression by *cyp6a17^MB^-Gal4>UAS-mCD8:GFP* (green). (A) *cyp6a17* was strongly expressed in the αβ lobe (white and open triangle) and γ lobe (white triangle) of the mushroom bodies. (B) The GFP expression was also detected in the ellipsoid bodies (EB, white arrowhead) and pars intercerebralis (PI, white and open arrowhead). (C) *cyp6a17^MB^*-*Gal4* derived GFP was expressed in the Kenyon cells (Kc) and Calyx (Ca) of the mushroom bodies. (D) A cross section of the peduncle shows GFP staining along the outer rim that may correspond to the γ lobe (red in Fig. 3E). It also shows weak circular staining in three ring structures that appear to be αβ lobes. Note that there is no staining inside the rings that may correspond to the αβ core. (E) Schematics of Figure (D). Red may correspond to axon fibers for γ lobe; blue for α′β′; gray for αβ; yellow for αβ core based on their characteristic position in the peduncle. (F) Rescue of *cyp6a17* mutant phenotypes by *cyp6a17^MB^-Gal4>UAS-cyp6a17*. Temperature preference profile and avoidance index (AI) of the mutant line expressing the *cyp6a17* transgene (*cyp6a17^Δ173^; cyp6a17^MB^-Gal4>UAS-cyp6a17*) are shown. Black bars, AI_High_; gray bars, AI_Low_. Two asterisks, *P*<0.001.

To detect *cyp6a17* expression, we crossed *cyp6a17^MB^-Gal4* transgenic flies to a reporter line *UAS-mCD8.GFP*. Confocal analysis of GFP expression in the adult brain revealed a complex pattern of expression in the mushroom bodies (Figure 3A). GAL4 is expressed in the mushroom body neurons that project to α-, β- and γ-lobes (Figure 3D & E), although it is not clear whether the *cyp6a17^MB^*-*Gal4* is expressed in all or a subset of mushroom body neurons. In addition to the mushroom bodies, GFP was also expressed in the ellipsoid bodies (EB), pars intercerebralis (PI) and several unknown lateral neurons (Figure 3B). Abnormal TPB phenotypes in the *cyp6a17^Δ173^* mutant was rescued by expressing wild-type *cyp6a17* using the *cyp6a17^MB^*-*Gal4* driver (AI_High_ = 0.74±0.03, *p*<0.001; AI_Low_ = 0.90±0.03) (Figure 3F and Table S2). These data suggest that the *cyp6a17^MB^*-*Gal4* reporter represents the pattern of endogenous *cyp6a17* expression.

### *cyp6a17* is required in the mushroom bodies for temperature preference behavior

**Figure 4:**
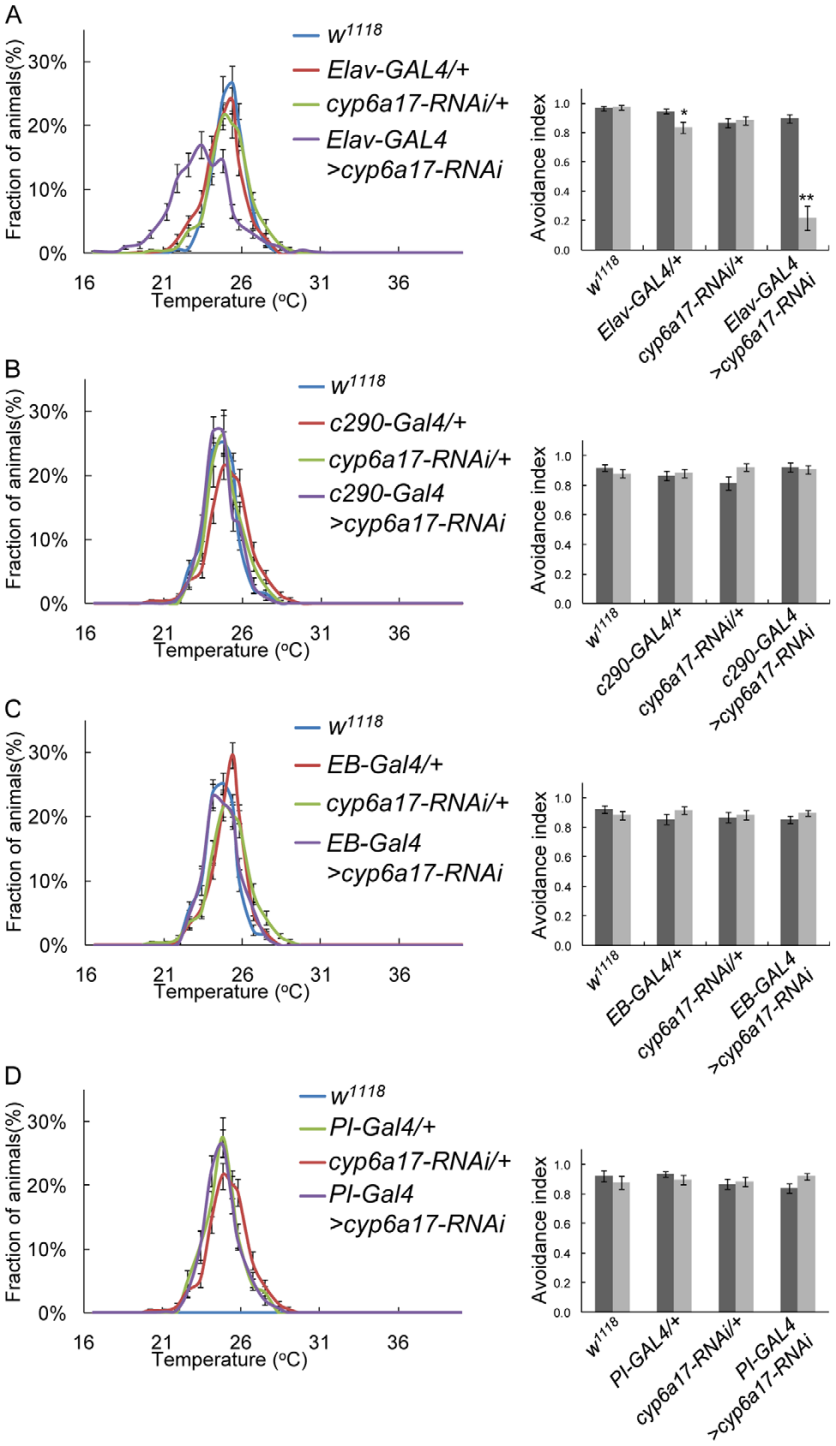
*cyp6a17* knockdown in the neurons results in abnormal temperature preference behavior. (A–D) Effects of tissue-specific knockdown of *cyp6a17* on TPB. *cyp6a17* was knockdowned by RNAi in pan-neuronal cell by *Elav*-*Gal4* (A), whole body except head *c290*-*Gal4* (B), ellipsoid bodies by *c232*-*Gal4 (EB-Gal4)* (C) and pars intercerebralis by *dilp2*-*Gal4* (*PI*-*Gal4*) (D). Temperature preference profile and avoidance index (AI) are shown. Black bars, AI_High_; gray bars, AI_Low_. One asterisks, *P*<0.005 and two asterisks, *P*<0.001.

To test whether *cyp6a17* is required for TPB in specific tissues, we examined the effects of reduced *cyp6a17* expression by targeted knockdown using *cyp6a17*-specific UAS-RNAi transgenic flies. To minimize potential off-target effects on other *Drosophila CYP* genes, we selected a relatively small unconserved region (222 base pairs) of *cyp6a17* as an RNAi target. Expression of *cyp6a17-RNAi* in the neuronal cells using a pan-neural *Elav-Gal4* driver resulted in a significant reduction of TPB (AI_High_ = 0.90±0.02; AI_Low_ = 0.22±0.14, *p*<0.001) (Figure 4A and Table S2). In contrast, *cyp6a17* RNAi expression in the whole body except the head by using *c290-Gal4* showed no effect on TPB (Figure 4B). These data suggest that *cyp6a17* function for normal TPB is required in neurons of the brain.

In the adult brain, *cyp6a17^MB^-Gal4* expression is enriched in the mushroom bodies as well as EB and PI (Figure 3A). *cyp6a17*-RNAi knockdown in subsets of EB and PI neurons labeled by *c232-Gal4* and *dilp2-Gal4*, showed little effect on TPB (Figure 4C & D and Table S2). This result is consistent with the previous report that EB and PI are not essential for TPB [8]. To exclude the possibility that *cyp6a17* RNAi affects locomotor activity, we examined the motor activity of the flies by climbing behavior test. In these control assays, locomotor activity was normal at all temperature conditions tested.

**Figure 5:**
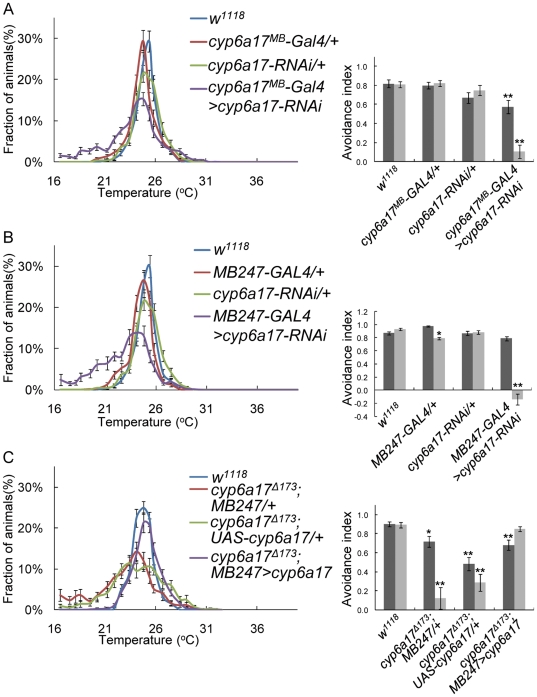
*cyp6a17* is required in the mushroom bodies for temperature preference behavior. (A–C) Effects of mushroom bodies-specific knockdown of *cyp6a17* on TPB. *cyp6a17* was knockdowned by RNAi in the *cyp6a17*-specific cells by *cyp6a17^MB^*-*Gal4* (A) or in the mushroom bodies by *MB247*-*Gal4* (B). (C) Rescue of *cyp6a17* mutant phenotype. Expression of *cyp6a17* transgene in the mushroom bodies by *MB247-Gal4* restored the *cyp6a17^Δ173^* phenotype. Black bars, AI_High_; gray bars, AI_Low_. One asterisks, *P*<0.005 and two asterisks, *P*<0.001.

**Figure 6:**
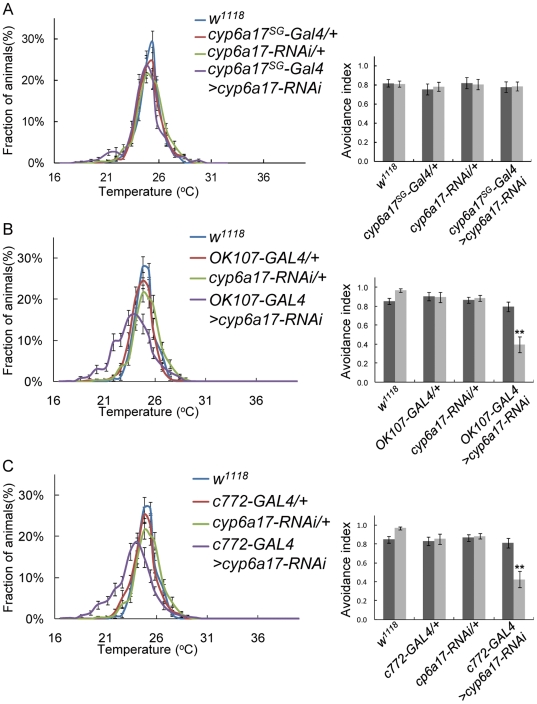
Effects of *cyp6a17* knockdown in surface glia cells and the mushroom bodies. (A) *cyp6a17* was knockdowned by RNAi in surface glia by *cyp6a17^SG^*-Gal4. (B–C) *cyp6a17* RNAi knockdown by two mushroom body-specific Gal4 lines, *OK107-Gal4*(B) and *c772-Gal4* (C), causes significant TPB defects in the distribution profiles and AI. Black bars, AI_High_; grey bars, AI_Low_. Two asterisks, *P*<0.001.

Next, we induced *cyp6a17-RNAi* in the *cyp6a17*-expressing cells by using *cyp6a17^MB^-Gal4*. These flies showed a reduced AI index, as seen in *cyp6a17* mutants (AI_High_ = 0.57±0.07, *p*<0.001; AI_Low_ = 0.11±0.07, *p*<0.001) (Figure 5A and Table S2). Thus, *cyp6a17* function is indeed required for TPB in the cells identified by the *cyp6a17* reporter. To determine whether *cyp6a17* is required in the mushroom bodies, we induced *cyp6a17*-RNAi preferentially in the mushroom bodies using *MB247-Gal4*. These flies with knockdown of *cyp6a17* in the mushroom bodies showed a reduced avoidance index in the TPB assay (AI_High_ = 0.89±0.03; AI_Low_ = 0.14±0.09, *p*<0.001) (Figure 5B and Table S2). In the heads of these flies, *cyp6a17* mRNA expression level was reduced compared with wild-type (0.56±0.06, *p*<0.001) (Figure S2A). *cyp6a17^MB^-Gal4* and MB247 show additional expression in surface glia [25]. To check whether *cyp6a17* in surface glia is required for TPB, we induced *cyp6a17-RNAi* in surface glia by *cyp6a17^SG^-Gal4* which drives strong Gal4 expression in surface glia but very weakly in the mushroom bodies (Figure S3). These flies showed normal TPB (Figure 6A and Table S2). In addition, *cyp6a17* RNAi knockdown by other mushroom body Gal4 lines like *OK107*- and *c772*-*Gal4* also resulted in slightly weaker but significant TPB defects: *OK107*-*Gal4* (AI_High_ = 0.80±0.02; AI_Low_ = 0.40±0.20, *p*<0.001) (Figure 6B and Table S2) and *c772*-*Gal4* (AI_High_ = 0.81±0.05; AI_Low_ = 0.43±0.13, *p*<0.001) (Figure 6C & Table S2). Furthermore, *cyp6a17* mutant phenotype was rescued by expressing the wild-type *cyp6a17* cDNA using the *MB247-Gal4* mushroom body driver (Figure 5C and Table S2). Taken together, these data suggest that *cyp6a17* expression in the mushroom bodies is necessary for normal low temperature TPB.

**Figure 7:**
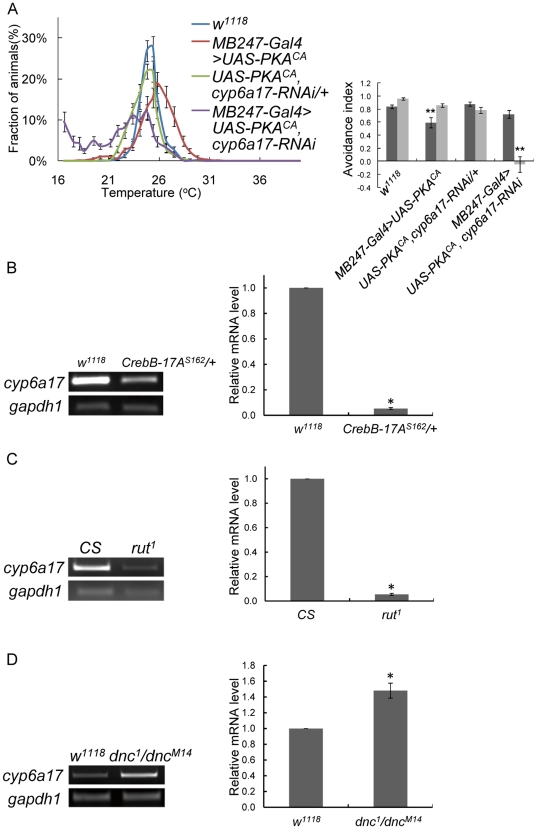
*cyp6a17* expression is regulated by PKA signaling pathway. (A) Temperature preference behavior of *MB247>UAS-PKA^CA^, cyp6a17-RNAi*. Temperature preference profile and avoidance index (AI) are shown. Black bars, AI_High_; gray bars, AI_Low_. Two asterisks, *P*<0.001. (B–D) Determination of expression level of *cyp6a17* in the PKA signaling pathway mutant background. *CrebB*-*17A^S162^* (B); *rut^1^* (C); and *dnc^M14^*/*dnc ^1^*(D). Relative transcript level was measured by real-time PCR (histogram). *gapdh1* was used as internal control. The number of tests: N = 3∼4. Asterisks, *P*<0.001 by t test. Error bars indicates S.E.M.

Previous studies proposed that the PKA signaling cascade is important for TPB [8]. According to our microarray data, *cyp6a17* expression is regulated by PKA in an activity-dependent manner. Thus we tested epistatic relationship between PKA and *cyp6a17*. It is noteworthy that TPB phenotypes induced by PKA^CA^ overexpression were strongly suppressed by *cyp6a17-RNAi* (AI_High_ = 0.72±0.06, *p* = 0.08; AI_Low_ = −0.05±0.12, *p*<0.001) (Figure 7A & Table S2). Furthermore, the transcript of *cyp6a17* was significantly decreased to 0.044±0.026 (*p*<0.001, t test) in *dCrebB*-*17A^s162^* mutant (Figure 7B). In *rut^1^*, which is a loss-of-function mutant for *rutabaga*, encoding calcium-calmodulin responsive adenylate cyclase, *cyp6a17* expression level was reduced compared with the wild-type level (0.054±0.008, *p*<0.001, t test) (Figure 7C). On the contrary, in the *dnc^1^/dnc^M14^* transheterozygote, which can increase cAMP level due to a defect in *Dunce* phosphodiesterase, *cyp6a17* expression level was slightly increased (1.48±0.05, *p*<0.001, t test) (Figure 7D). These results suggest that *cyp6a17* transcription is regulated by the cAMP-PKA signaling pathway and *cyp6a17* functions downstream to PKA for TPB.

## Discussion

Temperature preference is an important behavioral strategy for optimizing the body temperature in poikilothermal animals like *Drosophila*. TPB involves temperature sensing and processing of the sensory input at higher levels in the brain. Recent studies have implicated that PKA signaling in the mushroom bodies is required for TPB. However, it is largely unknown how PKA signaling pathway contributes to the behavioral response. In this study, we identified *cyp6a17* as a new gene regulated by PKA signaling. Our analysis provides several pieces of evidence that *cyp6a17* is required in the mushroom bodies for TPB. First, mutations in the *cyp6a17* gene showed reduced TPB. Second, abnormal TPB phenotypes in *cyp6a17* mutants were rescued with a wild-type *cyp6a17* transgene. Third, tissue-specific RNAi knockdown of *cyp6a17* expression in the mushroom bodies resulted in TPB defects.

TPB assays are measured by two indices, AI_Low_ and AI_High_, for avoiding low and high temperatures, respectively. It is interesting to note that loss of *cyp6a17* results in a preferential reduction of the AI_Low_ value compared with the AI_High_ score. This asymmetric effect of *cyp6a17* mutations suggests that *cyp6a17* might be selectively required for avoidance of low temperatures. We noted that the transgenic expression by *MB247>cyp6a17* from the *cyp6a17* mutant significantly lowers the AI_High_ index. However, one of the control strains for this test, *cyp6a17^Δ173^; UAS-cyp6a17/+*, shows a very low AI-High value in the absence of any Gal4 driver. Thus, the reduction of AI_High_ in this strain is unlikely to be due to *cyp6a17* overexpression. *cyp6a17* mutant and this transgene were outcrossed at least four times, but the effect of *UAS*-*cyp6a17*/+ in reducing the AI_High_ still remained. It is unknown why this strain shows non-specific low AI_High_. Importantly, however, transgenic overexpression of *Cyp6a17* in the presence of Gal4 restores the low AI_Low_ defect of the *cyp6a17^Δ173^* mutant (Fig. 5C). Previous studies have shown that increased levels of cAMP or PKA signaling cause stronger avoidance from low temperature whereas lower cAMP-PKA signaling levels result in the opposite trend with reduced avoidance. This is consistent with our data that *cyp6a17* mutants show lower AI_Low_. *cyp6a17* is necessary for TPB but may not be sufficient to induce AI_High_. Since *cyp6a17* is the only one of multiple downstream factors for PKA^CA^, it may need additional PKA downstream factors to induce AI_High_.

It has been reported that many cytochrome P450 proteins are expressed in the midgut, malpighian tubules and fat body whereas only one gene *cyp4g15* is detected in the brain of developing larvae [26]. We found that *cyp6a17* mRNA is expressed in the adult body including hindgut, but about 30% more expression is detected in the head (Figure S2B). Using a reporter construct regulated by a 5′ region of the *cyp6a17* gene, we showed that *cyp6a17* expression is highly enriched in the mushroom bodies, especially αβ and γ lobes. Three observations strongly support that this reporter reflects the pattern of *cyp6a17* gene expression. Firstly, *cyp6a17* RNAi driven by mushroom body-specific Gal4 lines causes TPB defects. Secondly, *cyp6a17* RNAi using *cyp6a17^MB^*-*Gal4* also results in similar TPB phenotypes. Thirdly, *cyp6a17* expression induced by *cyp6a17^MB^*-*Gal4* rescues *cyp6a17* null mutant phenotypes. These data indicate that the mushroom body regions labeled by *cyp6a17* reporter are the sites where *cyp6a17* function is required for TPB. To our knowledge, *cyp6a17* is the first gene of this family shown to be expressed in the mushroom bodies with specific function in TPB. However, it is possible that other cell types in addition to the mushroom bodies might also be involved in the TPB phenotype.

Cytochrome P450 proteins are known to participate in the synthesis of steroid hormone, thereby regulating homeostasis and thermal responses [27], [28]. Because *cyp6a17* is also expressed in PI and EB regions that have been implicated in neuroendocrine regulation [29], *cyp6a17* may play a role in the neuroendocrine system. However, it has been shown that the PI and EB are not required for TPB [8]. Furthermore, our data show that the *cyp6a17* mutant deficits in TPB are rescued by *cyp6a17* expression in the mushroom bodies but not in subsets of PI and EB neurons. Thus, it appears that the *cyp6a17* function for TPB is required mainly in the mushroom bodies rather than the PI and EB cells.

There are 83 CYP family genes in *Drosophila*, excluding 7 pseudogenes. Of these, only several CYP genes have been characterized for their expression patterns and functions. Some CYP proteins function together in a protein complex system for catalyzing oxidative reactions [30]. However, many CYP genes show tissue-specific expression profiles, suggesting that CYP genes may play distinct functions in diverse tissues. This possibility is supported by our finding that *cyp6a17* has a specific function in the mushroom bodies as a necessary component for controlling the temperature preference behavior. In addition to *cyp6a17*, other CYP genes might also play important roles in TPB.

cAMP-PKA signaling in the mushroom bodies is important for learning and memory in *Drosophila* [31], but downstream events are not well understood. Our study identified a cytochrome P450 gene as a downstream factor for PKA signaling in TPB. Given the potential relationship between temperature and stress related genes [32]–[34], this work could provide clues to understanding the relationship between stress response and temperature preference behavior. Although we focused on characterizing the function of *cyp6a17* in this study, our genome-wide screen identified many other genes that showed strong response to either PKA^CA^ or PKA^DN^ (Table S1). Further analysis of these genes might identify additional genetic factors involved in PKA-dependent TPB and their relationship with *cyp6a17*.

Until now, there is no report to our knowledge that *Drosophila* CYP is involved in temperature preference behavior. It is an intriguing question how PKA-dependent regulation of *cyp6a17* is functionally related to TPB. Since steroid hormone can modulate several kinds of behavior, neuronal homeostasis could be an important factor for TPB. Consistent with this idea, it has been shown that *Drosophila dystroglycan* plays a role in modulating energy homeostasis and the thermal responses [35]. Cyp6a17 is known to have monooxygenase activity [13]. Hence, it is plausible that *cyp6a17* might be involved in the synthesis of steroid hormones, thereby modulating temperature preference behavior. *cyp6a17* may also participate in the regulation of ATP level to modulate energy homeostasis in neurons. Detailed analysis of these possibilities in the future will help provide mechanistic insights into the function of *cyp6a17* for TPB.

## Materials and Methods

### *Drosophila* strains and transgenic lines

Fly stocks were raised on standard cornmeal food at 25°C and 40–50% relative humidity. *w^1118^* and *Canton-S* were used as wild-type strains. *UAS-PKA^DN^* and *PKA^CA^* were provided by D. Kalderon; other lines were provided as follows: *MB247, c772* (T. Zars), *c309* (L. C. Griffith), *MB247; tub-GAL80^ts^* (G. Roman), *P{SUpor-P}KG04448*, *c232*, *c290 and dilp2-Gal4* (Bloomington stock center). All mutant and transgenic alleles used in this study were outcrossed to the *w^1118^* line at least four times.

### Microarray analysis

Total RNA was extracted from adult heads. After extracting total RNA, microarrays were generated by Digital Genomics Inc. (Seoul, Korea). Modified cDNA from total RNA was hybridized to GeneChip® *Drosophila* Genome 2.0 Array. Image acquisition and data extraction from microarray experiments were analyzed by Affymetrix GeneChip® Scanner 3000 7G and Affymetrix GCOS1.4 software, respectively. Data were normalized by MAS5 and RMA (Robust Multi-array Average), and differentially expressed genes were selected by comparing control and transgenic lines. The functional category of selected genes was classified using the DAVID2007 (http://david.abcc.ncifcrf.gov/home.jsp) program.

### Temperature preference behavior assay and climbing assay

TPB assays were performed as described previously [8], [36]. 1–3 day old adult files of mixed gender were collected without anesthetization in vials containing fresh food during morning hours. After 3 days under a 12 hr light/dark incubation cycle, the collected flies were placed in the test device. A temperature gradient from 15 to 45°C with a slope of 0.75°C/cm was produced in an aluminum block (42 length×24 width×7 cm heights). Electronic thermal sensors were embedded in the block every 3 cm, and the gradient was established using cold and hot circulating water chambers at each end. A glass plate with five separate lanes was placed 2.5 mm above the block, creating suitable space for flies to walk. The glass plate was coated with quinine sulfate powder, an aversive stimulus for *Drosophila*, to prevent flies from escaping the temperature gradient by resting on the walls or roof of the lane. 1% yeast paste was applied on the block for flies to stay on the block. Adult flies were placed between the aluminum block and the glass plate, allowed to move for 25 min in the dark, and photographed with a digital camera with 2–5 msec exposure at the end of each assay. Avoidance indices against low temperature (AI_Low_) and high temperature (AI_High_) were calculated from the following formulae: AI_Low_ = N_Int_−N_Low_/N_Int_+N_Low_ and AI_High_ = N_Int_−N_High_/N_Int_+N_High_. N_Low_, N_High_ and N_Int_ are the numbers of flies in the region below 23.4°C, above 26.7°C, and between 23.4 and 26.7°C, respectively. A population of mixed sexes was tested for TPB. The number of flies was recorded by using digital photographs and processed with Microsoft Excel, and one-way ANOVA followed by Dunnett's post test was performed. All data points in the figures are represented as the mean and the standard error of the mean (s.e.m.), unless mentioned otherwise.

For climbing test to check the locomotor activity, groups of ten flies were transferred into vials and incubated for 1 hr at 25°C for environmental acclimatization. Then flies were transferred to 18°C and 32°C incubator for 15 min. After tapping the flies down to the bottom, we counted the number of flies that climb more than 10 cm from the bottom in 1 min. Three trials were performed for each genotype and repeated with 3 different batches. All mutant strains used in our TPB assays showed similar climbing rates as the wild-type. A horizontal locomotor activity test was done as described previously [37]. It showed that *cyp6a17^Δ173^* mutant flies are normal in the horizontal locomotor activity.

It has been reported that the mushroom bodies are involved in suppressing hyperactivity of flies [38]. We found that test flies of the same genotype placed at high and low temperatures show different locomotor activity at the beginning of the trial but results in the same distribution profiles at the end of the assay. This indicates that the difference in the initial locomotor activity does not affect the temperature preference behavior in the standard assay conditions that allow time for test flies to choose and settle at their preferred temperature.

### Construction of transgenic flies

*cyp6a17-RNAi* construct was generated as described [39]. *cyp6a17-RNAi* flies contain two copies of a 222 bp fragment of *cyp6a17* cDNA. PCR primers used to create the target region for *cyp6a17* knockdown were 5′- TGCTCTAGACAGCTTGTACGATCCAAA-3′ and 5′- TGCTCTAGAAATCATTTCGCTTTTCCT-3′. A PCR fragment was cloned into pWIZ vector. To construct *UAS-cyp6a17*, 1.5 kb fragment of a *cyp6a17* cDNA obtained from the *Drosophila* Genome Research Center was cloned into pUAST. A *cyp6a17-Gal4* construct was generated using pCaSper vector and 1,201 bp sequence upstream from the transcription start site of the *cyp6a17* gene. *cyp6a17^SG^-Gal4* was inserted at 78A1. Unlike *cyp6a17^MB^*, Gal4 expression in the mushroom bodies was strongly repressed, thus showing a preferential expression in the surface glia. To make a *cyp6a17* genomic rescue construct, a 4.2 kb *SacI*-digested genomic fragment containing about 1.2 kb upstream from the transcription start site and about 1.5 kb downstream from the transcription stop site, was cloned into pCaSper vector. Transgenic lines were generated in the *w^1118^* background using standard germline transformation techniques.

### Relative quantitative RT-PCR

4–6 day old adult flies were collected and frozen immediately in liquid nitrogen. Heads were removed from bodies by vortex-mixing and sorting through a chilled sieve. Total RNA was extracted from heads with a Trizol (Invitrogen) and reverse transcribed using the AMV reverse transcriptase system (Promega). RT-PCR was performed with the following primers: for *cyp6a17*, 5′-GTTGTTACTGGCGCTAATCGT-3′ and 5′-TTCCATCACAACCTGCTCCA-3′; for *cyp6a22*, 5′-TTCGGGAAACTGTGAAGCAG-3′ and 5′-GATAAAAACATTTGATTGTGT-3′; for *cyp6a23*, 5′-TCGCTGTTACTAACGTTGAT-3′ and 5′-TTTCCATGACGACCTGCTCCA-3′; for *gapdh1*, 5′-ACCGTCGACGGTCCCTCT-3′ and 5′-GTGTAGCCCAGGATTCCCT-3′. Real-time PCR was performed with iCycler iQ (Bio-Rad) using the KAPA SYBR FAST Bio-Rad iCycler qPCR kit (Kapa Biosystems). The level of *gapdh1* mRNA was measured as an internal control for the RNA amount in each sample. Data processing was performed with Microsoft Excel. All quantitative analysis were done more than three times.

### Immunohistochemistry

Adult brains expressing transgenic *cyp6a17-Gal4>UAS-mCD8.GFP* were removed from head capsules and fixed in 4% paraformaldehyde in PBS for 30 min, and rinsed in PBS containing 0.5% Triton X-100. For staining with anti-*FasII* (Hybridoma Bank), adult brains were fixed for 30 min in 4% paraformaldehyde in PBS containing 0.5% Triton X-100. Fixed samples were washed for 10 min three times and incubated with 5% normal goat serum. Antibody dilution was 1∶50 for mouse anti-*FasII* and 1∶250 for goat anti-mouse Alexa Fluor 568 (Molecular Probe). Confocal analysis was performed on a Zeiss LSM710 microscope. Confocal stacks were processed with ZEN 2009 Light Edition and Adobe Photoshop.

## Supporting Information

Figure S1 **Effects on** ***cyp6a17*** **mutation on mushroom body morphology.** Mushroom body morphology in *cyp6a17^Δ173^* mutant. Mushroom body axons are visualized using *FASII* antisera. Compared with wild type (A), no significant defects were found in mutant (B). Doral is up.
(PDF)

Figure S2 **Effects of** ***cyp6a17*** **RNAi and tissue distribution of** ***cyp6a17*** **expression.** (A) Reduction of the expression level by *MB247>cyp6a17-RNAi*. *gapdh1* was used as internal control. The number of tests: N = 4. Two asterisks, *P*<0.001. (B) Transcriptional profiles of *cyp6a17* in the head and body assayed by real-time PCR. The histogram shows that *cyp6a17* mRNA is slightly more abundant in the head compared with the body. *gapdh1* was used as internal control. The number of tests: N = 4. Two asterisks, *P*<0.001. All data are means, and the error bars indicate s.e.m.
(PDF)

Figure S3 **The expression pattern of** ***cyp6a17^SG^*** **-** ***Gal4*** **.** *cyp6a17* reporter expression by *cyp6a17^SG^-Gal4>UAS-mCD8:GFP* (green). GFP expression was detected strongly in surface glial cells (filled triangle) and weakly in Kenyon cells (open triangle).
(PDF)

Table S1 **Total list of the 103 probes.**
(PDF)

Table S2 **Statistical analysis of the results shown in Figures.**
(PDF)
