# Supplementary figures and images for: Novel Cytochrome P450, cyp6a17, Is Required for Temperature Preference Behavior in Drosophila
Source: PLoS One. 2011 Dec 28;6(12):e29800. doi: 10.1371/journal.pone.0029800 (PMC3247289; doi:10.1371/journal.pone.0029800)

Supporting Information Figure 1

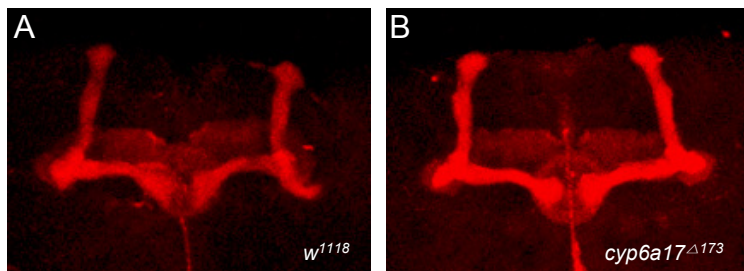

Supplement: Figure S1 — Effects on cyp6a17 mutation on mushroom body morphology. Mushroom body morphology in cyp6a17Δ173 mutant. Mushroom body axons are visualized using FASII antisera. Compared with wild type (A), no significant defects were found in mutant (B). Doral is up. (PDF) [file pone.0029800.s001.pdf]

Supporting Information Figure 2

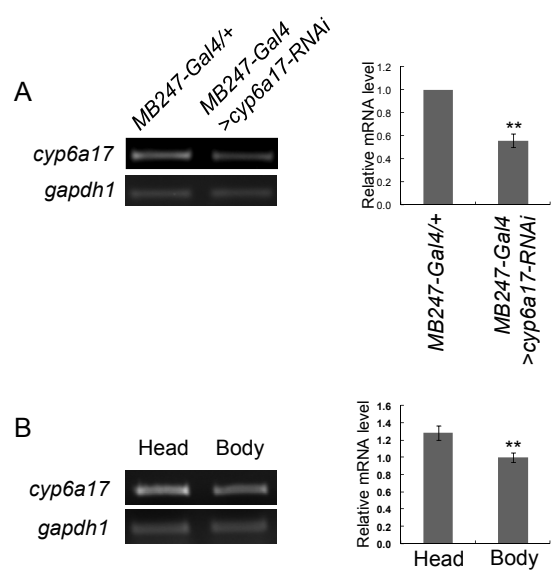

Supplement: Figure S2 — Effects of cyp6a17 RNAi and tissue distribution of cyp6a17 expression. (A) Reduction of the expression level by MB247>cyp6a17-RNAi. gapdh1 was used as internal control. The number of tests: N = 4. Two asterisks, P<0.001. (B) Transcriptional profiles of cyp6a17 in the head and body assayed by real-time PCR. The histogram shows that cyp6a17 mRNA is slightly more abundant in the head compared with the body. gapdh1 was used as internal control. The number of tests: N = 4. Two asterisks, P<0.001. All data are means, and the error bars indicate s.e.m. (PDF) [file pone.0029800.s002.pdf]

Supporting Information Figure 3

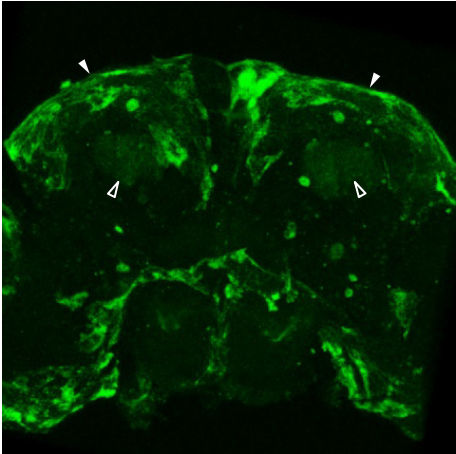

Supplement: Figure S3 — The expression pattern of cyp6a17SG - Gal4 . cyp6a17 reporter expression by cyp6a17SG-Gal4>UAS-mCD8:GFP (green). GFP expression was detected strongly in surface glial cells (filled triangle) and weakly in Kenyon cells (open triangle). (PDF) [file pone.0029800.s003.pdf]
