# Supplementary material for: Novel Cytochrome P450, cyp6a17, Is Required for Temperature Preference Behavior in Drosophila
Source: PLoS One. 2011 Dec 28;6(12):e29800. doi: 10.1371/journal.pone.0029800 (PMC3247289; doi:10.1371/journal.pone.0029800)
Supplement: Table S1 — Total list of the 103 probes. (PDF) [file pone.0029800.s004.pdf]

Supporting Table 1. Total list of the 103 probes.

| Gene Symbol | Fold value more than 2 compare with control | | Functional category |
|---|---|---|---|
| | MB247; tub-Gal80$^{ts}$ >UAS-PKA$^{DN}$ | MB247; tub-Gal80$^{ts}$ >UAS-PKA$^{CA}$ | |
| CG4650 | 0.05462545 | 0.18353308 | Proteolysis |
| CG13646 | 0.38492279 | 0.33395972 | Amino acid transport |
| CG7763 | 0.37176426 | 0.33785872 | N/A |
| CG8713 | 0.17261539 | 0.34251213 | Potassium ion transport |
| CG8193 | 0.38644473 | 0.37035790 | Oxygen transporter |
| Bc | 0.32859148 | 0.39365999 | Melanization defense response |
| CG7738 | 0.45168221 | 0.42349006 | N/A |
| Eip74EF | 0.35456597 | 0.47670544 | Transcription factor |
| CG11852 | 2.06968183 | 2.04899718 | N/A |
| Acp1 (CG31904) | 3.12055794 | 2.08231164 | Structural constituent of adult cuticle |
| p38c | 2.09828406 | 2.43528395 | MAP kinase |
| CG32850 | 2.07906087 | 2.44630621 | Zinc ion binding |
| CG7214 | 3.52039424 | 2.69286704 | Regulation of NFAT |
| Cyp6a17 | 0.20840532 | 3.03124613 | Monooxygenase |
| CG31685 | 3.03643187 | 3.11841298 | N/A |
| CG10924 | 4.58314522 | 4.86540533 | Gluconeogenesis |
| CG11893 | 9.92378858 | 7.62364531 | N/A |
| CG2177 | 10.06949900 | 10.55403670 | Metal ion transport |
| Transposon.32 | 6.56581454 | 14.01341819 | Transposon |
| Transposon.17 | 8.91080024 | 14.91172237 | Transposon |
| path | 0.23115653 | 1.16722040 | Regulation of growth |
| CG31038 | 0.26355738 | 1.70542700 | N/A |
| CR31614 | 0.28676304 | 0.54815432 | N/A |
| CG33296 | 0.29712584 | 0.65189703 | Neurotransmitter transport |
| CG9512 | 0.35926966 | 0.65928310 | Alcohol metabolic process |
| CG31809 | 0.40768337 | 0.75573639 | Steroid metabolic process |
| pgant4 | 0.43227970 | 0.52068365 | Polypeptide N-acetylgalactosaminyl transferase |
| regucalcin | 0.43480216 | 0.79394667 | Lipid particle |
| TotA | 0.44192701 | 0.88571840 | Response to stress |
| CG2781 | 0.44634557 | 0.99414693 | Fatty acid elongation |
| HDC07874 | 0.46297494 | 0.69483484 | RNAi reagent |
| Amph | 0.46755857 | 1.04774018 | Neurotransmitter secretion |
| Os-C | 0.47353468 | 0.53434707 | Pheromone binding |
| CG32364 | 0.47360723 | 0.56594745 | Nucleic acid binding |
| CG3513 | 2.07621234 | 1.23572096 | Endopeptidase inhibitor |
| Cyp9c1 | 2.08830871 | 0.85557071 | Monooxygenase |
| Ssl2 | 2.09409806 | 0.87558309 | Strictosidine synthase |
| Rab2 | 2.13104639 | 1.04633440 | GTPase |
| Edg91 | 2.19929632 | 1.07025902 | Structural constituent of pupal cuticle |
| Def | 2.20113861 | 0.96875382 | Defense response to bacterium |
| CG8012 | 2.22890789 | 1.61419067 | N/A |
| CecC | 2.23797667 | 0.87502128 | Defense response to bacterium |
| CG33868 | 2.28082854 | 1.57578969 | Chromatin remodeling |
| α-Est2 | 2.35839603 | 1.60646105 | Carboxylesterase |
| neb | 2.40444012 | 0.93318434 | Chromosome segregation |
| Su(var)3-3 | 2.45636849 | 1.68926071 | Histone demethylation |
| CG5172 | 2.52585006 | 1.00000000 | N/A |
| Cyp6a8 | 2.56855862 | 0.78826521 | Insecticide metabolic process |
| CG8539 | 2.59846387 | 1.37374097 | Proteolysis |
| CG14221 | 2.68433923 | 1.79533616 | Cell redox homeostasis |
| AttC | 2.75668419 | 1.68841787 | Antibacterial humoral response |
| dro4 | 2.76519649 | 0.84640145 | Defense response to fungus |
| Glut1 | 3.20877360 | 0.91934483 | Regulation of cell cycle |
| CG1942 | 3.39646738 | 1.31813305 | Mesoderm development |
| CG17567 | 3.46586870 | 1.00952735 | N/A |
| DptB | 3.75399050 | 1.66270243 | Antibacterial humoral response |
| CG32594 | 4.14788817 | 1.57232342 | N/A |

Supporting Table 1. Total list of the 103 probes. (continued)

| Gene Symbol | Fold value more than 2 compare with control | | Functional category |
|---|---|---|---|
| | MB247; tub-Gal80$^{ts}$ >UAS-PKA$^{DN}$ | MB247; tub-Gal80$^{ts}$ >UAS-PKA$^{CA}$ | |
| Snoo | 4.18018560 | 0.97805873 | Neuron development |
| dro5 | 4.25088138 | 0.88446012 | Defense response to fungus |
| CG10148 | 4.44437022 | 0.91550204 | Protein binding |
| AttA | 11.38347400 | 1.20780370 | Antibacterial humoral response |
| AttB | 12.10004913 | 1.56159896 | Antibacterial humoral response |
| CG5246 | 14.87449723 | 1.45160507 | Proteolysis |
| mthl6 | 27.13174625 | 1.30304237 | G-protein coupled receptor |
| Cyp4g1 | 0.68625373 | 0.14760311 | Lipid metabolic process |
| CG4757 | 0.79106053 | 0.31502146 | Carboxylesterase |
| Cyp12d1-d (Cyp12d1-p) | 1.14747363 | 0.32020528 | Monooxygenase |
| CG18343 | 1.62984639 | 0.33558822 | N/A |
| Cyp6a2 | 1.07802677 | 0.35180186 | Response to caffeine and insecticide |
| CG6234 | 0.56344160 | 0.37776816 | N/A |
| CG13022 | 1.20618316 | 0.38462595 | N/A |
| CG18673 | 1.65334638 | 0.39260569 | Carbonate dehydratase |
| CG13091 | 0.63889422 | 0.39279651 | Metabolic process |
| CG13428 | 0.56814024 | 0.40542698 | N/A |
| CG4446 | 0.77331227 | 0.40591709 | N/A |
| CG3790 | 0.59197945 | 0.42939938 | Carnitine transporter |
| e | 0.57661809 | 0.44989742 | β-alanyl-dopamine synthase |
| CG40485 | 1.40489859 | 0.45862058 | Oxidoreductase |
| CG6639 | 1.04399587 | 0.46297558 | Proteolysis |
| CG31809 | 0.50393061 | 0.46758936 | Steroid metabolic process |
| CG7194 | 1.20777189 | 2.01424476 | Gonad development |
| Obp83cd | 1.12733946 | 2.02309771 | Sensory perception of chemical stimulus |
| CG33640 | 1.01327376 | 2.02588180 | N/A |
| CG9837 | 1.15903168 | 2.04624515 | N/A |
| Ugt37c1 | 1.38855180 | 2.04678703 | Glucuronosyltransferase |
| Ilp5 | 1.24536617 | 2.11140304 | Insulin receptor signaling pathway |
| copiaGIP | 1.30491877 | 2.13132411 | DNA integration |
| Lsp1β | 1.25239530 | 2.13392578 | Nutrient reservoir |
| Transposon.35 | 1.46128340 | 2.16903450 | Transposon |
| CG31157 | 0.98758033 | 2.31018278 | N/A |
| CG8757 | 0.97552517 | 2.32367290 | Oxidation reduction |
| CG17189 | 1.16356112 | 2.32393546 | N/A |
| CG11425 | 1.32017392 | 2.41124626 | Phosphatidate phosphatase |
| MtnD | 1.09222342 | 2.41458793 | Metal ion binding |
| CG13641 | 1.22003286 | 2.47338530 | N/A |
| Obp99a | 1.76759653 | 2.47462342 | Response to pheromone |
| CG10962 | 1.54358080 | 2.53717143 | Oxidation reduction |
| Transposon.39 | 0.97334692 | 2.58391390 | Transposon |
| ninaD | 0.70583310 | 2.71650744 | Phototransduction |
| CG10962 | 1.58207281 | 2.77948000 | Oxidation reduction |
| Prx2540-2 | 1.81008695 | 2.98099096 | Hydrogen peroxide catabolic process |
| Transposon.75 | 1.73781198 | 4.24100740 | Transposon |
| Hsc70-2 | 1.03142291 | 4.93773017 | Response to heat |

Red indicates up-regulation in the experimental sample, and blue represents down-regulation compare to control transgenic flies (*MB247-Gal4; tub-Gal80$^{ts}$/+*). N/A means not available.
