# Supplementary material for: Novel Cytochrome P450, cyp6a17, Is Required for Temperature Preference Behavior in Drosophila
Source: PLoS One. 2011 Dec 28;6(12):e29800. doi: 10.1371/journal.pone.0029800 (PMC3247289; doi:10.1371/journal.pone.0029800)
Supplement: Table S2 — Statistical analysis of the results shown in Figures. (PDF) [file pone.0029800.s005.pdf]

Supporting Table 2. Statistical analysis of the results shown in Figures.

Figure	Genotype	N(n)	Average temperature(℃) ± SD	Comparing with control			
				AI_{Low} ± s.e.m.	$P[AI_{Low}]$	AI_{High} ± s.e.m.	$P[AI_{High}]$
2C	*w^{1118}*	21 (1001)	25.2 ± 0.3	0.9860 ± 0.01	control	0.8564 ± 0.03	control
	$cyp6a17^{rev}$	17(749)	25.0 ± 0.5	0.8882 ± 0.04		0.8397 ± 0.03	
	$cyp6a17^{\Delta173}$	17 (719)	24.1 ± 2.0	0.5612 ± 0.06	**	0.9042 ± 0.03	
2D	*w^{1118}*	16 (754)	25.1 ± 0.3	0.9270 ± 0.03	control	0.8564 ± 0.03	control
	$cyp6a17^{GR}$	17 (1023)	25.1 ± 0.6	0.9270 ± 0.02		0.7856 ± 0.04	
	$cyp6a17^{\Delta173}$	17 (719)	24.1 ± 2.0	0.5612 ± 0.06	**	0.9042 ± 0.03	
	$cyp6a17^{\Delta173}$; $cyp6a17^{GR}$	16 (666)	24.8 ± 0.5	0.8751 ± 0.03		0.8408 ± 0.05	
3F	*w^{1118}*	18 (828)	24.8 ± 0.4	0.8926 ± 0.03	control	0.9026 ± 0.03	control
	$cyp6a17^{\Delta173}$	17 (719)	24.1 ± 2.0	0.5612 ± 0.06	**	0.9042 ± 0.03	
	$cyp6a17^{\Delta173}$;$cyp6a17^{MB}$-Gal4 >UAS-cyp6a17	15 (579)	25.1 ± 0.6	0.9005 ± 0.03		0.7377 ± 0.03	**
4A	*w^{1118}*	15 (723)	25.2 ± 0.3	0.9724 ± 0.01	control	0.9656 ± 0.01	control
	Elav-Gal4/+	24 (1071)	24.8 ± 0.6	0.8333 ± 0.04		0.9453 ± 0.01	
	cyp6a17-RNAi/+	18 (783)	25.1 ± 0.7	0.8827 ± 0.03		0.8655 ± 0.03	*
	Elav>cyp6a17-RNAi	21 (952)	23.7 ± 1.6	0.2181 ± 0.08	**	0.8967 ± 0.03	
4B	*w1118*	16(723)	24.7 ± 0.4	0.8813 ± 0.03	control	0.9193 ± 0.03	control
	c290-Gal4/+	15 (555)	24.9 ± 0.5	0.9243 ± 0.03		0.8132 ± 0.04	
	cyp6a17-RNAi/+	18 (783)	25.1 ± 0.7	0.8827 ± 0.03		0.8655 ± 0.03	
	c290-Gal4>cyp6a17-RNAi	15 (549)	24.6 ± 0.4	0.9080 ± 0.03		0.9207 ± 0.03	
4C	*w1118*	16 (723)	24.7 ± 0.4	0.8813 ± 0.03	control	0.9193 ± 0.03	control
	EB-Gal4/+	21 (1020)	25.1 ± 0.4	0.8537 ± 0.04		0.9142 ± 0.02	
	cyp6a17-RNAi/+	18 (783)	25.1 ± 0.7	0.8827 ± 0.03		0.8655 ± 0.03	
	EB-Gal4>cyp6a17-RNAi	20 (704)	24.8 ± 0.5	0.8977 ± 0.02		0.8533 ± 0.02	
4D	*w1118*	16(723)	24.7 ± 0.4	0.8813 ± 0.03	control	0.9193 ± 0.03	control
	PI-Gal4/+	15 (596)	24.8 ± 0.5	0.8952 ± 0.03		0.9315 ± 0.02	
	cyp6a17-RNAi/+	18 (783)	25.1 ± 0.7	0.8827 ± 0.03		0.8655 ± 0.03	
	PI-Gal4>cyp6a17-RNAi	16 (685)	24.8 ± 0.5	0.9187 ± 0.02		0.8375 ± 0.03	

One way ANOVA followed by Dunnett's tests were performed. N, Number of testes. n, number of flies tested. SD, standard deviation, s.e.m., standard error. One asterisks, *P*<0.005 and two asterisks, *P*<0.001.

Supporting Table 2. Statistical analysis of the results shown in Figures (continued).

Figure	Genotype	N(n)	Average temperature(℃) ± SD	Comparing with control			
				AI_{Low} ± s.e.m.	P[AI_{Low}]	AI_{High} ± s.e.m.	P[AI_{High}]
5A	*w1118*	28 (1350)	25.0 ± 0.2	0.9270 ± 0.03	control	0.8178 ± 0.04	control
	cyp6a17^{MB}*-Gal4/+*	21 (832)	24.7 ± 0.8	0.8234 ± 0.03		0.7985 ± 0.03	
	cyp6a17-RNAi/+	18 (783)	25.1 ± 0.7	0.8827 ± 0.03		0.8655 ± 0.03	
	cyp6a17^{MB}*-Gal4>cyp6a17-RNAi*	23 (1100)	23.9 ± 3.2	0.1057 ± 0.07	**	0.5742 ± 0.07	**
5B	*w*^{1118}	28 (956)	25.0 ± 0.4	0.9270 ± 0.02	control	0.8707 ± 0.02	control
	MB247/+	23 (854)	24.6 ± 0.6	0.7917 ± 0.04		0.9716 ± 0.01	*
	cyp6a17-RNAi/+	18 (783)	25.1 ± 0.7	0.8827 ± 0.03		0.8655 ± 0.03	
	MB247>cyp6a17-RNAi	21 (736)	23.0 ± 2.7	0.1446 ± 0.09	**	0.8874 ± 0.03	
5C	*w*^{1118}	18 (828)	24.8 ± 0.4	0.8926 ± 0.03	control	0.9026 ± 0.03	control
	cyp6a17$^{\Delta 173}$*; MB247-Gal4/+*	16 (769)	23.1 ± 2.9	0.1235 ± 0.11	**	0.7170 ± 0.05	*
	cyp6a17$^{\Delta 173}$*; UAS-cyp6a17/+*	15 (691)	24.2 ± 2.9	0.2855 ± 0.09	**	0.4812 ± 0.07	**
	cyp6a17$^{\Delta 173}$*; MB247-Gal4 /UAS-cyp6a17*	19 (956)	25.1 ± 0.9	0.8503 ± 0.02		0.6813 ± 0.05	**
6A	*w*^{1118}	24 (1093)	25.1 ± 0.4	0.8102 ± 0.03	control	0.8178 ± 0.04	control
	cyp6a17^{SG}*-Gal4/+*	16 (678)	25.1 ± 0.6	0.7817 ± 0.05		0.7537 ± 0.06	
	cyp6a17-RNAi/+	18 (783)	25.1 ± 0.7	0.8827 ± 0.03		0.8655 ± 0.03	
	cyp6a17^{SG}*-Gal4>cyp6a17-RNAi*	20 (840)	24.7 ± 1.0	0.7846 ± 0.05		0.7770 ± 0.06	
6B	*w*^{1118}	14 (682)	25.0 ± 0.4	0.9646 ± 0.02	control	0.8508 ± 0.03	control
	OK107-Gal4/+	18 (944)	24.8 ± 0.5	0.8943 ± 0.04		0.9027 ± 0.03	
	cyp6a17-RNAi/+	18 (783)	25.1 ± 0.7	0.8827 ± 0.03		0.8655 ± 0.03	
	OK107>cyp6a17-RNAi	21 (943)	23.9 ± 1.3	0.3964 ± 0.10	**	0.7970 ± 0.03	
6C	*w*^{1118}	15 (735)	25.0 ± 0.4	0.9670 ± 0.02	control	0.8458 ± 0.03	control
	c772-Gal4/+	15 (743)	24.8 ± 0.5	0.8533 ± 0.05		0.8303 ± 0.04	
	cyp6a17-RNAi/+	18 (783)	25.1 ± 0.7	0.8827 ± 0.03		0.8655 ± 0.03	
	c772>cyp6a17-RNAi	19 (1009)	23.9 ± 1.5	0.4251 ± 0.08	**	0.8108 ± 0.05	
7A	*w*^{1118}	19 (764)	25.1 ± 0.4	0.9585 ± 0.02	control	0.8359 ± 0.03	control
	MB247>UAS-PKA^{CA}	16(737)	25.7 ± 1.3	0.8599 ± 0.03		0.5899 ± 0.08	**
	UAS-PKACA, cyp6a17-RNAi/+	20 (846)	24.6 ± 7.0	0.7793 ± 0.04		0.8774 ± 0.03	
	MB247>UAS-PKA^{CA} *cyp6a17-RNAi/+*	22 (1057)	22.2 ± 3.5	-0.0469 ± 0.12	**	0.7200 ± 0.06	

One way ANOVA followed by Dunnett's tests were performed. N, Number of testes. n, number of flies tested. SD, standard deviation, s.e.m., standard error. One asterisks, $P<0.005$ and two asterisks, $P<0.001$.
